# Cervical Intraepithelial Neoplasia Grade 3 in the Third Trimester of Pregnancy: A Case Report

**DOI:** 10.7759/cureus.46703

**Published:** 2023-10-09

**Authors:** Laksh S Agrawal, Shazia Mohammad, Neema Acharya, Shreyash Huse

**Affiliations:** 1 Internal Medicine, Jawaharlal Nehru Medical College, Datta Meghe Institute of Higher Education and Research, Wardha, IND; 2 Obstetrics and Gynaecology, Jawaharlal Nehru Medical College, Datta Meghe Institute of Higher Education and Research, Wardha, IND; 3 Medicine, Jawaharlal Nehru Medical College, Datta Meghe Institute of Higher Education and Research, Wardha, IND

**Keywords:** hpv-dna, pregnancy trimester third, human papillomavirus, high risk pregnancy, cervical intraepithelial neoplasia (cin)

## Abstract

The incidence of malignancies during pregnancy has been on the rise in the recent years, primarily due to an increase in older age pregnancies. This poses a significant risk to both the mother and the developing fetus. We present the case of a 29-year-old woman who experienced intermittent vaginal bleeding during her pregnancy. In the last trimester, the patient presented with abnormal vaginal bleeding and abdominal pain. The gestational age was 37.6 weeks. Notably, to our knowledge,* *there have been no reported cases of grade 3 cervical intraepithelial neoplasia in the third trimester.

## Introduction

The incidence of gynecological malignancies is steadily increasing, and cervical cancer during pregnancy poses a threat to both the fetus and the mother. Cervical intraepithelial neoplasia (CIN) and cervical cancer are among the most common cytological diagnoses in pregnant women and are significant public health concerns [1]. Pregnancy, higher hormone levels, and immunosuppression are the risk factors for cervical human papillomavirus (HPV) infection and viral proliferation [2]. While Pap smear (cytology) is the conventional screening method for cervical malignancy, HPV DNA testing has shown greater sensitivity and a higher negative predictive value, making it an alternative criterion for screening [3]. Cervical malignancy is associated with advanced age and inadequate spacing between children. Researchers have also observed a correlation between hormone levels (estrogen, progesterone, and human chorionic gonadotropin) during pregnancy and the presence of high-risk HPV types 16 and 18, indicating a potential acceleration of cervical cancer development during pregnancy [4]. In this report, we present the case of a 29-year-old woman with a gestational age of 37.6 weeks who presented with complaints of abnormal vaginal bleeding.

## Case presentation

Patient information

A 29-year-old woman was brought to the emergency department with complaints of vaginal bleeding. She was at a gestational age of 37.6 weeks and was a primigravida (G1). The abnormal vaginal bleeding was irregular in nature and there was no purulent vaginal discharge present. There was no family history of malignancy. The patient did not undergo any Pap smear. On bimanual examination, the vulva was soft and stretchable. Effacement was observed up to the cervix, with a dilation of 2 cm extending up to the internal os. The vagina shows increased vascularity and soft, moist, and bluish distension. The cervix and vagina were healthy.

**Figure 1:**
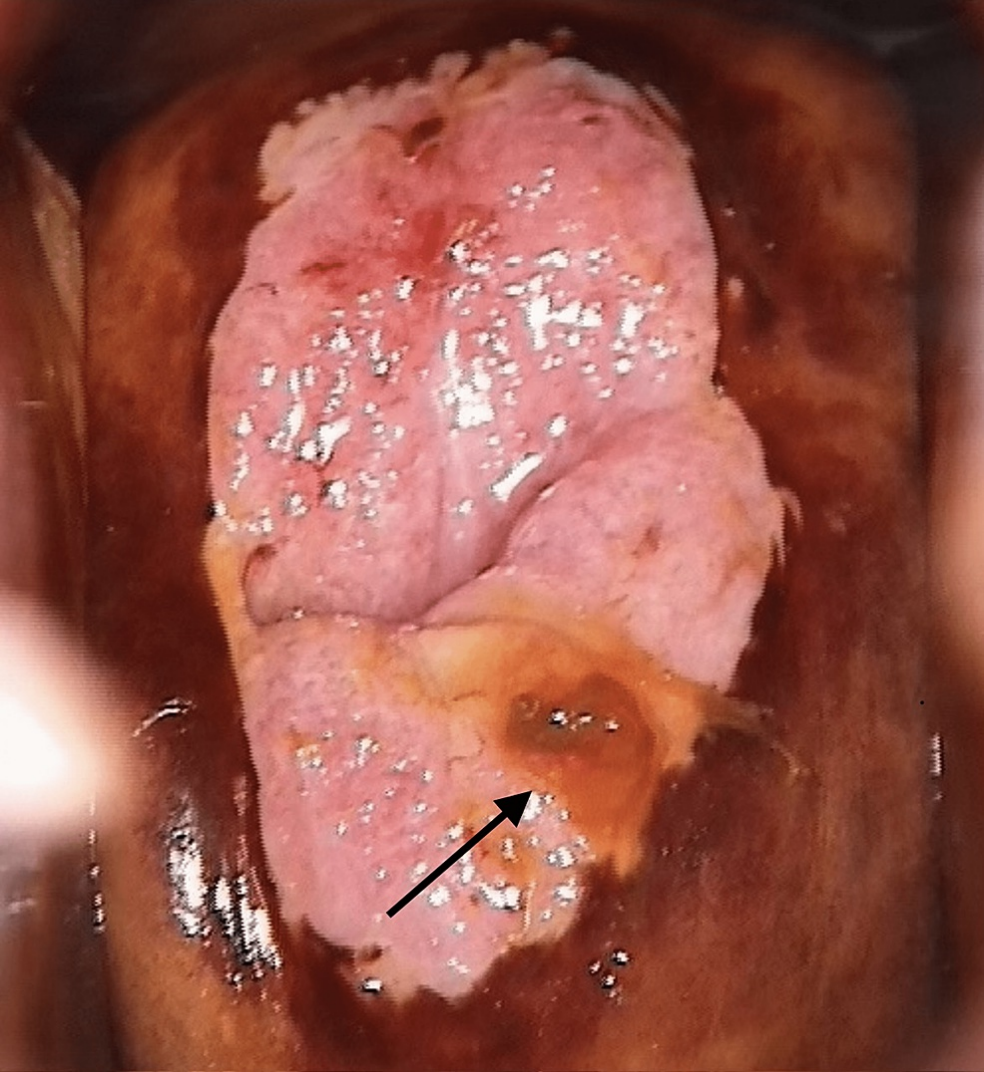
Colposcopy image showing a lesion 6 mm in length and 3-4 mm in width, via visual inspection with Lugol's iodine (VILI)

HPV DNA testing was performed, identifying several major types, including 16, 18, 31, 33, 39, and 68, as well as minor types 6 and 11. The most commonly found high-risk HPV types were 16 and 18. A colposcopy was conducted to rule out malignancy, and it was scored 7 according to the Swede score grading system. The gold standard method for confirming the diagnosis is a Pap smear (cytology). Colposcopy images revealed a lesion measuring approximately 6 mm in length and 3-4 mm in width (Figure 1).

Clinical findings

After examination, she was transferred for vaginal delivery. The delivery occurred spontaneously without any associated systemic abnormalities. During the vaginal examination, bleeding was observed. Intravenous fluids and injectable ceftriaxone were initiated for her treatment.

Intervention

In this patient, the diagnostic method considered was the HPV DNA test, which was performed and repeated every six weeks. The patient was kept under observation. As she was near term, spontaneous labor was identified as the preferred treatment option. At 38 weeks and 6 days, the patient underwent spontaneous labor. She was advised to refrain from oral intake (nil per oral) as she was about to deliver. Vital signs were monitored throughout the process. The woman successfully delivered a male child weighing 2.8 kg. Both the intrapartum and postpartum periods were uneventful. She was advised to maintain follow-up visits every three weeks for a total of 12 weeks postpartum. A repeat colposcopy and cytology procedure were scheduled for the same. We asked her to return for a follow-up appointment 12 weeks postpartum, during which her repeat colposcopy and cytology would be assessed and further management could be planned.

## Discussion

Cervical cancer is one of the most prevalent malignancies during pregnancy, with an estimated incidence of 0.8 to 1.5 cases per 10,000 live births [4]. In rural parts of India, CIN during pregnancy is a commonly encountered medical emergency. India has a maternal mortality rate of 26.619 deaths per 1000 live births [5]. Infection with HPV, which has been found in up to 99% of women with squamous cervical cancer, serves as the initial event in the development of cervical dysplasia and carcinogenesis [5]. The majority of HPV infections are acquired in the first few years following sexual debut and tend to spontaneously clear, leading to a rapid decline in infection rates thereafter [2].

There are various grades of neoplasia associated with HPV subtypes 16 and 18. The most common grade encountered during pregnancy is CIN 1, which is considered a low-risk type [5]. It often manifests as spotting per vagina in the second trimester. Surgical intervention during the late stages of pregnancy can be challenging. Pregnancy, due to elevated hormone levels and immunosuppression, appears to be a risk factor for developing cervical HPV infection and increased viral replication [6]. The treatment course for CIN is determined by the gestational age at the time of diagnosis. Alternatively, tumors can be monitored, or pregnancy can be interrupted for definitive treatment according to established standards. The Pap smear is an essential component of early antenatal care as it allows for the early detection of cervical cancer. In cases of advanced pregnancy, colposcopy is the preferred method for detecting neoplasia, as hormone-related cellular changes can be misdiagnosed through Pap smears. Colposcopy is a safe and reliable procedure that can be conducted during pregnancy.

## Conclusions

In conclusion, cervical cancer during pregnancy represents a rare and potentially life-threatening emergency. Early detection and timely treatment are vital for the well-being of both the mother and the fetus as the progression is slow and the management is dependent on the gestational age. Effective management of cervical cancer during pregnancy can be achieved through proper screening and intervention. This underscores the significance of early identification and prompt treatment. All women above 35 years of age including pregnant women should prioritize a healthy lifestyle and undergo regular cervical cancer screenings to ensure early detection and appropriate management. Further research is required to determine the most optimal screening and treatment approaches for cervical cancer during pregnancy.
